# Supplementary material for: Electrical Control of the Transduction Channels’ Gating Force in Mechanosensory Hair Cells
Source: Adv Sci (Weinh). 2026 Jan 14;13(16):e09171. doi: 10.1002/advs.202509171 (PMC13042940; doi:10.1002/advs.202509171)
Supplement: Supplementary file 1 — Supporting File 1: advs73703‐sup‐0001‐SuppMat.pdf. [file ADVS-13-e09171-s002.pdf]

Supporting Information

Electrical control of the transduction channels' gating force in sensory hair cells

Achille Joliot[1], Laure Stickel[1] and Pascal Martin[1,]*

[1] Physique des Cellules et Cancer, Institut Curie, Université PSL, Sorbonne Université, CNRS UMR168, F-75005 Paris, France.

[*] Correspondence: pascal.martin@curie.fr

The supplementary information includes:

Tables S1 and S2

Figures S1 to S3

Legends for Movies S1 to S3

Other supporting information for this manuscript includes the following:

Movies S1 to S3

Supplementary Table

Table S1 Parameter values with 95% intervals for fits of force-displacement relations with Equation 1.

Variable	Definition	Fig. 2A		Fig. 2B		Fig. 2C		Fig. 2D		Fig. 7E	
I (μA)	Transepithelial current	0	+3	0	+6	0	−3	0	−6	n/a	n/a
I_{IONTO} (nA)	Iontophoretic current	n/a	n/a	n/a	n/a	n/a	n/a	n/a	n/a	+10	−60
X_0 (nm)	Set-point deflection	−3 ± 2	1 ± 1	1 ± 2	+6 ± 4	−0.2 ± 0.6	−12 ± 1	−2 ± 3	n/a	−14 ± 3	n/a
K $(\mathrm{pN \cdot nm^{-1}})$	Stiffness	0.96 ± 0.04	0.91 ± 0.02	0.83 ± 0.03	0.78 ± 0.02	0.61 ± 0.02	0.66 ± 0.03	0.82 ± 0.04	0.96 ± 0.02	0.75 ± 0.07	0.80 ± 0.01
F_G (pN)	Gating force	21 ± 3	13 ± 2	18 ± 3	9 ± 3	19 ± 2	36 ± 3	22 ± 5	n/a	22 ± 7	n/a
δ (nm)	Characteristic length	4 ± 2	2 ± 2	2 ± 7	5 ± 4	9 ± 1	13 ± 1	6 ± 4	n/a	7 ± 4	n/a
$F_{\emptyset}$ (pN)	Force constant	14 ± 2	5 ± 1	7 ± 2	0.6 ± 1	10 ± 1	25 ± 1	11 ± 2	n/a	18 ± 4	n/a
R^2	Coefficient of determination	0.999	0.994	0.986	0.986	0.986	0.986	0.986	0.986	0.979	0.993

Table S2 Statistical significance and power in two-tailed paired Student's t-test.

	Fig. 5F		Fig. 6C		Fig. 7D	
**Number of replicates**	n = 8 cells in 6 frogs		n = 4 cells in 3 frogs		n = 8 cells in 6 frogs	
**Variable**	Max. friction force, ϕ_{MAX} [pN]		Rise time, τ_{10-90} [ms]		Max. friction force, ϕ_{MAX} [pN]	
**Test**	Start (1) vs Pre-drop (2)	Start (1) vs Pre-drop (2)	Onset:$\phi_{MAX}(1)$ vs $\bar{X}$ (2)	Offset:$\phi_{MAX}(1)$ vs $\bar{X}$ (2)	Start (1) vs Pre-drop (2)	Start (1) vs Post-drop (2)
**Mean (1)**	10.2	10.2	180	412	10.1	10.1
**SD (1)**	1.7	1.7	55	118	1.3	1.3
**Mean (2)**	13.2	12.1	487	100	12.1	8.2
**SD (2)**	2.2	1.6	64	44.1	1.6	1.19
**Effect size**	1.7	1.6	5.6	−2.6	1.6	−1.4
**Statistical power**	0.98	1.00	1.00	0.92	0.97	0.92
**p-value**	$2 \cdot 10^{-2}$	$1.5 \cdot 10^{-2}$	$1.5 \cdot 10^{-2}$	$1.5 \cdot 10^{-2}$	$1.5 \cdot 10^{-2}$	$5 \cdot 10^{-3}$

Supplementary Figures

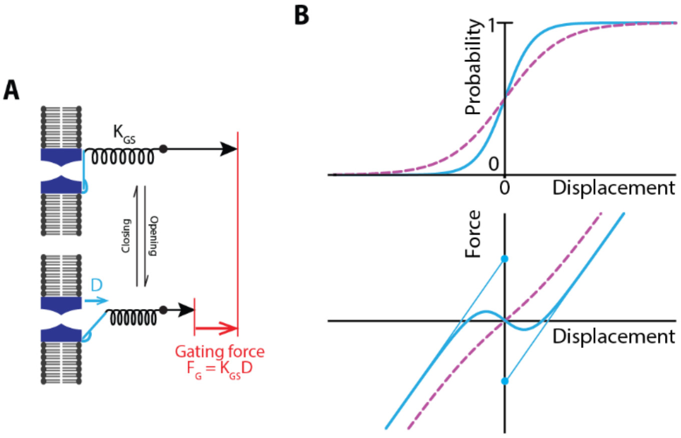

Figure S1 : Gating-spring model of mechanoelectrical transduction. **(A)** The concept of gating force. When the transduction channels switch between their open and closed state, tension in the gating springs is reduced (black arrows); this mechanical correlate of channel gating is called the gating force, $F_G = K_{GS}D$ (red arrow), where K_{GS} is the combined stiffness of all the gating springs operating in parallel in the hair bundle and D is the gating swing of each channel. **(B)** Electrical and mechanical responses to hair-bundle displacement. Top: sigmoidal relationship of channel open probability to displacement in the case of weak (dashed magenta line) and strong (solid blue line) gating force. The larger the gating force, the steeper the sigmoid and the more sensitive the hair cell is to displacement of its hair bundle. Bottom: The force–displacement relationship displays a shallow nonlinearity when sensitivity is low (dashed magenta curve) and a strong nonlinearity when sensitivity is high (smooth blue curve). The gating force is given by the vertical shift between the two linear limbs of the force-displacement relation; it is here shown in the case of high sensitivity as the distance between the two disks.

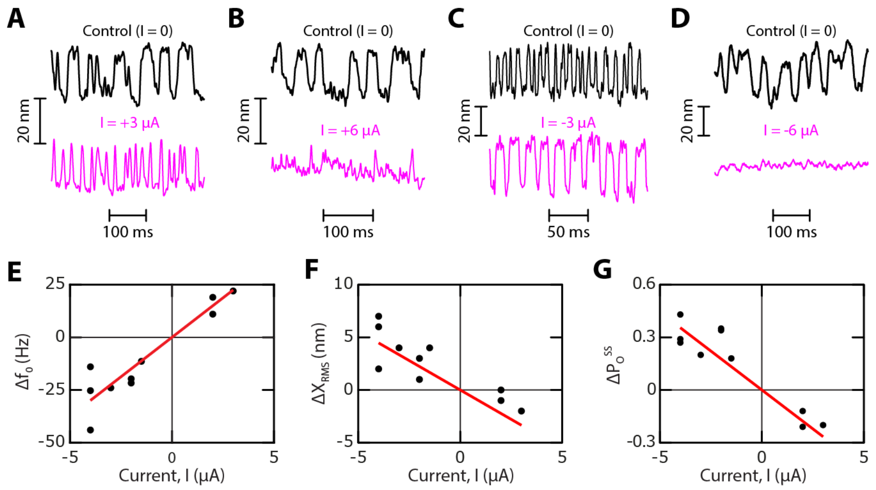

Figure S2 : Effects of a transepithelial current on spontaneous hair-bundle oscillations. Under control conditions ($I = 0$), over an ensemble of $n = 10$ oscillatory hair bundles, the oscillations had a characteristic frequency $f_0 = 22 \pm 14$ Hz, a root-mean-squared (RMS) amplitude $X_{RMS} = 13 \pm 4$ nm and their bimodal position histograms were associated with a steady-state open probability for the transduction channels $P_O^{SS} = 0.4 \pm 0.1$, thus close to $1/2$. **(A-D)** Representative examples of spontaneous hair-bundle oscillations under control conditions ($I = 0$; top) and in the presence of a transepithelial current (bottom) $I = +3$ µA (A), $I = +6$ µA (B), $I = -3$ µA (C), and $I = -6$ µA (D). **(E)** The change, Δf_0, of the oscillation frequency displayed a strong positive correlation to the transepithelial current (Pearson correlation $r = +0.9$; p-value = 10^{-4}; $n = 10$). **(F)** The change, ΔX_{RMS}, of the RMS amplitude of spontaneous oscillations displayed a strong negative correlation to the transepithelial current (Pearson correlation $r = -0.9$; p-value = 1×10^{-3}; $n = 10$). **(G)** The change, ΔP_O^{SS}, of the open probability at steady state displayed a strong negative correlation to the transepithelial current (Pearson correlation r = -0.9, p-value = 1×10^{-4}, $n = 10$). In (A), the transepithelial current evoked an increase of the oscillation frequency from 17 Hz to 39 Hz, a decrease of the RMS amplitude of oscillation from 7 nm to 5 nm and a decrease of the steady-state open probability from 0.5 to 0.3. Conversely in (B), the frequency of oscillation decreased from 49 Hz to 25 Hz, the RMS amplitude of oscillation increased from 11 nm to 15 nm and the steady-state open probability increased from 0.4 to 0.6. In the presence of a large positive current ($I = +6$ µA; panel B), the hair bundle only produced irregular spiky movements in the positive direction: the transduction channels were closed most of the time and evinced brief openings. Instead, a large negative current ($I = -6$ µA; panel D) completely abolished spontaneous oscillations. Red lines in (E-G) correspond to proportional fits of respective slopes 7 Hz $\cdot$ µA^{-1} ($R^2 = 0.86$; panel E), -1.1 nm $\cdot$ µA^{-1} ($R^2 = 0.6$; panel F), and -0.08 µA^{-1} ($R^2 = 0.83$; panel G).

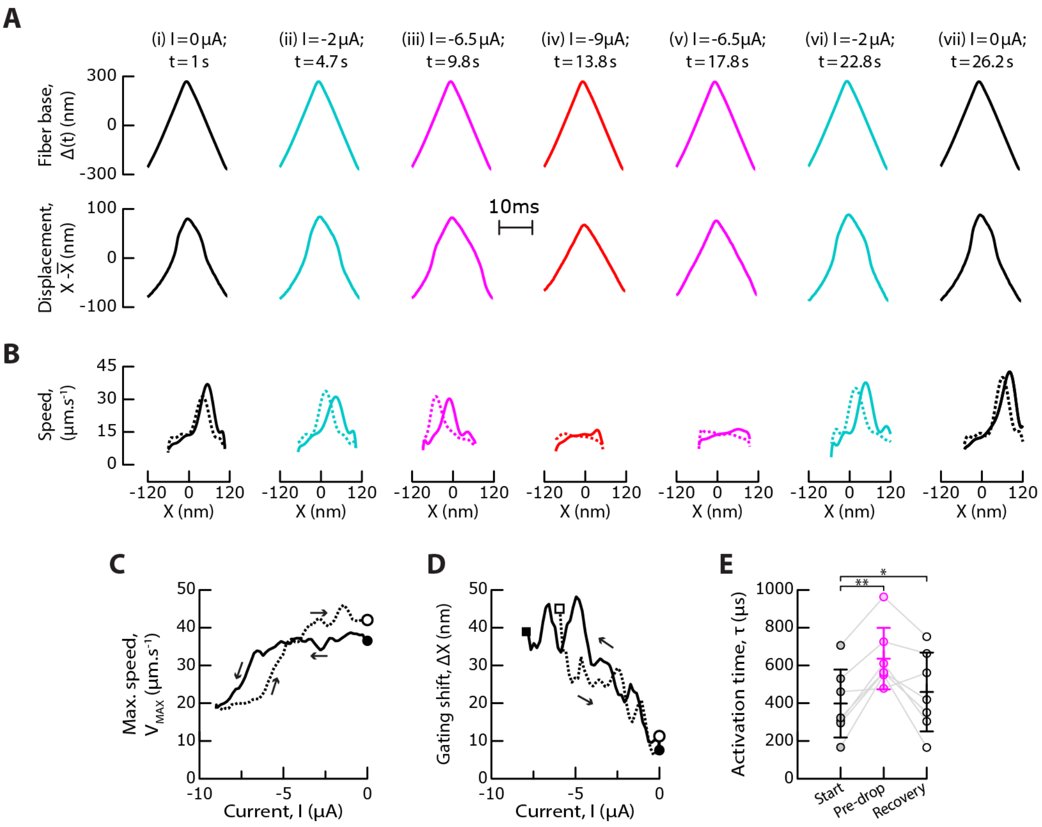

Figure S3 : Effect of a transepithelial current on the channel-activation time. Same data as in Figure 5A-E, to which this figure is associated. The time course of successive ramps of descending and ascending transepithelial current can be found in Figure 5A. **(A)** Fiber-base displacement, Δ (*top*), and hair-bundle displacement, X (*bottom*), as a function of time for one stimulus cycle at time points and values of the transepithelial current indicated at the top of each time trace. **(B)** Hair-bundle speed, $|dX/dt|$, on the positive (*solid line*) and negative (*dashed line*) half cycles of mechanical stimulation as a function of displacement, X. The distance between the two peaks, ΔX, defines the gating shift. **(C-D)** Maximum speed, V_{MAX} (C), and gating shift, ΔX (D), as a function of the transepithelial current, I, which varied in time from start (black disk) to finish (white disk). Arrows indicate the direction of circulation along the curve. Solid and dashed lines correspond to the descending and ascending ramps of transepithelial currents, respectively. In panel (D), a black square indicates the current at which the signatures of gating compliance and gating friction vanished on the descending ramp; bellow this current, the gating shift is no longer defined. Conversely, a white square indicates recovery of the signatures of gating compliance and gating friction on the ascending ramp. **(E)** For the same ensemble of hair bundles as in Figure 5, the distributions of the estimated activation time of the transduction channel, $\tau = \Delta X/(2\,V_{MAX})$, are shown at the start of the experiment ($I = 0$, black), before the drop of the maximal friction force (magenta, see Figure 5F), and at the end of the experiment ($I = 0$, white). Error bars: mean $\pm$ SD. Statistical significance was assessed using a paired t-test with, from p-values from left to right: $0.0014, 0.021$ *p-value $\leq$ 0.05, **p-value $\leq$ 0.01.

Legends for supplementary movies

Movie S1 (separate file): Transition between states of strong and weak gating force. Movie associated to Figure 5A-E. The force-displacement cycle in response to triangular mechanical stimulation of the hair bundle (top) is shown over time as the transepithelial current follows successive descending and ascending ramps (bottom). The friction force, $\phi(X)$, at each displacement X of the hair bundle is color coded as indicated on the right; it is given by the half-height of the force-displacement cycle. To obtain smooth videos, the force-displacement cycles were here averaged over 15 cycles, instead of 10 cycles in the data shown in Figure 5.

Movie S2 (separate file): Kinetics of the transition between states of strong and weak gating force. Movie associated to Figure 6A-B. The force-displacement cycle in response to triangular mechanical stimulation of the hair bundle (top) is shown over time before, during and after application of a negative step of transepithelial current (bottom). The friction force, $\phi(X)$, at each displacement X of the hair bundle is color coded as indicated on the right; it is given by the half-height of the force-displacement cycle. The force-displacement cycles were here averaged over 5 cycles.

Movie S3 Transition between states of strong and weak gating force upon a decrease of extracellular calcium concentration. Movie associated to Figure 7A-D. The force-displacement cycle in response to triangular mechanical stimulation of the hair bundle (top) is shown over time as an iontophoretic current trough a pipette containing a calcium chelator and positioned near a hair bundle follows successive descending and ascending ramps (bottom). The friction force, $\phi(X)$, at each displacement X of the hair bundle is color coded as indicated on the right; it is given by the half-height of the force-displacement cycle. To obtain smooth videos, the force-displacement cycles were here averaged over 15 cycles, instead of 10 cycles for the data shown in Figure 7.
